# Supplementary material for: Evaluating the effectiveness of data governance frameworks in ensuring security and privacy of healthcare data: A quantitative analysis of ISO standards, GDPR, and HIPAA in blockchain technology
Source: PLoS One. 2025 May 23;20(5):e0324285. doi: 10.1371/journal.pone.0324285 (PMC12101661; doi:10.1371/journal.pone.0324285)
Supplement: Attachment — Submitted filename: Response to Reviewers.docx [file pone.0324285.s003.docx]

To

The Academic Editor

PLOS ONE

Dear Dr Shadab Alam,

First of all, we, the authors are most grateful to the anonymous referees for their constructive and helpful comments that helped to improve the content and presentation of the paper, considerably. We have carefully noted the reviewers’ feedback and made appropriate changes to each comment in this revised manuscript. We feel confident that the quality of the manuscript has been significantly improved.

The following table provides details of our responses to the comments received by the editor and two reviewers.

Thank you.

Ameer Ahmed

**Editor comments**

| **Comment (Originally taken from the feedback letter)** | **Response - All changes are underlined in the revised manuscript. The page and line numbers for the changes refer to the MS Word-generated pages and lines on the revised manuscript.** |
| --- | --- |
| 1. Please ensure that your manuscript meets PLOS ONE's style requirements, including those for file naming. The PLOS ONE style templates can be found at  <https://journals.plos.org/plosone/s/file?id=wjVg/PLOSOne_formatting_sample_main_body.pdf> and  <https://journals.plos.org/plosone/s/file?id=ba62/PLOSOne_formatting_sample_title_authors_affiliations.pdf> | Thank you very much for bringing our attention to this point. We have now reviewed the formatting style mentioned in the links and have adopted it throughout the main document.  ***(Throughout the Manuscript)*** |
| PLOS requires an ORCID iD for the corresponding author in Editorial Manager on papers submitted after December 6th, 2016. Please ensure that you have an ORCID iD and that it is validated in Editorial Manager. To do this, go to ‘Update my Information’ (in the upper left-hand corner of the main menu), and click on the Fetch/Validate link next to the ORCID field. This will take you to the ORCID site and allow you to create a new iD or authenticate a pre-existing iD in Editorial Manager | Thank you for pointing out the requirement of ORCID iDs. As advised, it is ensured that the corresponding author has an ORCID iD and pre-existing ORCID in ‘Update my Information’ (in the upper left-hand corner of the main menu) has been updated/linked. |
| Both reviewers have recommended major revisions, highlighting concerns related to the manuscript; please address these thoroughly to enhance the manuscript's quality | Thank you for bringing our attention to these very important and significant points. All the points mentioned in the corresponding left column have been understood and addressed to enhance the manuscript's quality.  ***(Throughout the Manuscript)*** |

**Reviewer 1**

| **Comment (Originally taken from the feedback letter)** | **Response - All changes are underlined in the revised manuscript. The page and line numbers for the changes refer to the MS Word generated pages and lines on the revised manuscript.** |
| --- | --- |
| The introduction is disorganized, with several unnecessary sentences. The primary goal needs rewriting. | Thank you for bringing this point to our attention. The primary goal has now been rewritten more clearly. Moreover, major amendments have been made in the introduction section keeping in view the following points:-   - Added detailed discussion, integrating various aspects of blockchain applications in healthcare, covering both technical aspects and challenges. It provides additional background information before defining the research objective. - Made more elaborative by discussing blockchain’s working principles (e.g., proof-of-work, smart contracts, cryptographic hashes) and its specific applications in healthcare. - For flow and connectivity, the ideas have now been presented more clearly, discussing blockchain’s general advantages before narrowing it down to healthcare applications. - For technical depth, discussed blockchain's mechanics, such as cryptographic hashing, smart contracts, and consensus mechanisms, in more depth. - Reformulates the question slightly with a grammatical inconsistency: “Are existing data governance frameworks i.e. ISO standards, GDPR, and HIPAA effective in ensuring the security and privacy of healthcare data?”. - We made the introduction more narrative, however, concise, by providing a discussion on the gap in blockchain applications in Healthcare, before narrowing it down to research objectives.   ***(Introduction)*** |
| The literature review lacks a systematic approach and needs significant revision. consider a narrative style and use subheadings | Thank you for mentioning this important shortcoming in our previous version of the manuscript. As advised, a **systematic approach** and **narrative style** were adopted by a combination of clear subheadings, logical sequencing, referenced literature, real-world examples, and critical analysis. This makes the discussion **well-organized, academic, and insightful** while being accessible to readers. The following was considered:-  **Systematic Approach**   - The passage progresses from a broad introduction to specific aspects of healthcare data governance, creating a clear, methodical development of ideas. - Each section builds upon the previous one, ensuring coherence and depth in argumentation. - The subheadings ("Blockchain in Healthcare," "Data Governance Frameworks," "Challenges in Low-Resource Settings," etc.) help segment the discussion into distinct, focused areas. - This categorization makes the text easier to navigate and allows each section to address a specific component of the broader topic. - The passage references multiple studies and authors (e.g., "Swan, 2015," "Mougayar, 2016") to support claims, ensuring academic rigour. - The passage contrasts different frameworks and theories, such as Nissenbaum's privacy theory vs. data governance challenges in low-resource settings, showing a structured, comparative discussion.   **Narrative Style**   - The passage starts with an introduction to the issue, then presents existing governance models, followed by challenges in low-resource settings, and finally, emerging technologies and ethical considerations. This logical progression maintains a narrative flow. - The passage discusses both the strengths and limitations of existing governance models, ensuring a nuanced perspective. For example, it acknowledges the presence of privacy frameworks (e.g., GDPR, HIPAA) but also critiques their limitations in decentralized and IoT-driven healthcare. - The mention of Royal Free Trust and Alphabet’s DeepMind Health illustrates real-world conflicts between AI-driven healthcare innovation and regulatory constraints, making the discussion relatable. - Instead of merely listing information, the passage critiques existing governance models and advocates for more robust, adaptive frameworks. - The concluding section reinforces the need for governance models that integrate ethical, technical, and organizational considerations, adding to the narrative momentum.   ***(Literature Review)*** |
| The survey methodology is unclear, particularly regarding the rationale for participant distribution. The classification of "other" is ambiguous and needs further explanation | Thank you very much for bringing our attention to the shortcomings in the methodology section. We have now added more details in the methodology section regarding the survey and rationale for participant distribution which are as follows:-  “*The research focused on participants from Islamabad, Lahore, and Karachi, chosen for their diverse demographic profiles, advanced healthcare facilities, and varying levels of technology adoption. These cities represented both developed and developing health systems, allowing for a comprehensive assessment of data governance effectiveness in different contexts. A total of 250 participants were selected to ensure statistical validity, maintaining a 95% confidence level with a 5% margin of error while also considering practical limitations regarding participant availability. The participant pool included healthcare professionals, IT specialists, blockchain developers, administrators, and researchers, all of whom were directly involved in implementing and sustaining blockchain-based healthcare data platforms.*  Moreover, as advised, The classification of "other" has now been clearly explained as follows:  *“****Additionally, the 'other' category encompassed data analysts, compliance officers, and healthcare policy advisors, broadening the study’s perspective on governance structures.*** *To ensure balanced representation across various organizational roles and levels within the healthcare industry, a stratified random sampling* *technique was employed. This approach strengthened the findings by incorporating diverse viewpoints from different professional backgrounds and organizational hierarchies.”*  ***(Methodology)*** |
| The conclusions drawn are vague and largely based on conjecture. Clear lessons learned should be explicitly stated. | Thank you for mentioning this point: As advised, To effectively address vagueness, we have provided concrete examples, such as MedRec and Estonia's eHealth Foundation, to illustrate blockchain’s role in healthcare data governance. These case studies clarify how blockchain aligns with frameworks like GDPR and HIPAA, making the discussion more tangible. Additionally, the research findings are compared against existing literature, specifically mentioning Mettler (2016) and Winter & Davidson (2017), to show how blockchain enhances compliance in ways traditional frameworks cannot fully achieve. The discussion also acknowledges blockchain’s limitations, such as the challenge of GDPR's "right to be forgotten," and suggests hybrid solutions like off-chain storage or cryptographic techniques to mitigate these issues.  Moreover, as advised, the lessons learned are now explicitly mentioned below:-  **“Lessons Learned**   - ***Blockchain enhances compliance*** *– The study shows that blockchain improves adherence to HIPAA by ensuring security and patient authorization through encryption and smart contracts.* - ***Policy adaptation is necessary*** *– The analysis highlights the need for ongoing updates to regulatory frameworks to align with evolving technologies like blockchain.* - ***Sector-specific frameworks benefit the most*** *– While ISO and GDPR provide general governance principles, HIPAA’s sectoral focus makes it better suited for blockchain integration* - ***Hybrid solutions bridge regulatory gaps*** *– The research suggests that technical solutions like off-chain storage help reconcile blockchain’s immutability with GDPR’s data deletion requirement”*   ***(Discussion)*** |
| The manuscript lacks a dedicated limitations section, which is essential for transparency and scientific rigor | Thank you very much for mentioning this point. As recommended, a detailed limitation section has been added to the manuscript as follows:  **“*Sample Size & Geographic Bias***  *The study had a small sample size, mainly healthcare professionals and IT experts from specific regions, limiting the generalizability of findings. Geographic bias may affect the applicability of results to different regulatory environments. Future research should include broader, more diverse samples for global insights.*  ***Response Bias in Survey Tool***  *Self-reported survey data may have been influenced by social desirability or limited familiarity with regulatory frameworks, leading to biased perceptions. This could impact the validity of findings on data governance effectiveness. Future studies should incorporate triangulation methods, such as interviews and observations, to enhance accuracy.*  ***Limited Scope of Data Governance Standards***  *The study focused only on ISO, GDPR, and HIPAA, overlooking other relevant or emerging frameworks. It did not consider technologies like blockchain and AI, which impact healthcare data governance. Future research should explore a wider range of standards for a more comprehensive perspective.*  ***Need for Larger, Methodologically Diverse Studies***  *A larger sample and diverse research methods are needed to strengthen evidence and practical relevance. Incorporating qualitative and mixed-method approaches would provide deeper insights. Future studies should aim for a more holistic understanding of healthcare data governance frameworks”.*  ***(Limitations)*** |
| Lastly, several grammatical errors are present throughout the manuscript and need to be addressed  . | Thank you for bringing our attention to the grammatical errors. These have been carefully removed throughout the manuscript. Also, we engaged a copyeditor who thoroughly reviewed the manuscript and helped in addressing the comments.  ***(Throughout the Manuscript)*** |

**Reviewer 2**

| **Comment (Originally taken from the feedback letter)** | **Response - All changes are underlined in the revised manuscript.** |
| --- | --- |
| The manuscript is well-structured and provides a clear overview of the research problem, methodology and findings. However, in the introduction section, more concise details about the research gap and objectives are required. | Thank you for bringing this point to our attention. We have now made some major amendments in the introduction section by adding concise details about the research gap and objectives. For example, the following has now been added.  **Research Gap:**  *“Despite the growing interest in blockchain for healthcare data management, there remains a significant gap in understanding how blockchain can be effectively integrated with existing data governance frameworks such as ISO standards, GDPR, and HIPAA. These frameworks, while comprehensive, may struggle to accommodate the decentralized nature of blockchain and might not fully address emerging security and privacy challenges in healthcare data governance. Limited research exists on whether these regulatory standards are adequate to safeguard healthcare data within a blockchain-driven ecosystem*”  **Research Objective:**  The primary objective of this research is to evaluate the effectiveness of current data governance models—ISO standards, GDPR, and HIPAA—in ensuring the privacy and security of healthcare data when implemented alongside blockchain technology.  Specifically, the study aims to answer:  ***“Are current data governance models (i.e., ISO standards, GDPR, and HIPAA) adequate in providing security and privacy for healthcare data in a blockchain environment?”***  ***(Introduction)*** |
| The study addresses an important aspect i.e. blockchain technology in healthcare. However, manuscript could better emphasize its unique contributions to the existing literature. On one hand comparison of GDPR, HIPAA and ISO is valuable, the integration of Blockchain technology as a potential solution could be valuable if there is more focus on findings and discussion | Thank you very much for bringing our attention to this point. A one-hand comparison of GDPR, HIPAA, and ISO is valuable because it highlights their similarities and differences in regulating healthcare data privacy, security, and governance. Each framework has a distinct focus:  **GDPR** (General Data Protection Regulation): Enforces strict data privacy and consent mechanisms across the EU, emphasizing patient rights, data minimization, and cross-border data sharing regulations.  **HIPAA** (Health Insurance Portability and Accountability Act): Primarily focuses on protecting patient health information (PHI) within the U.S., ensuring security and confidentiality through compliance requirements for covered entities.  **ISO Standards** (e.g., ISO/IEC 27001): Provide internationally recognized guidelines for information security management, offering a structured framework adaptable to various industries, including healthcare.  Our study makes a unique contribution by comparing these frameworks and suggesting the strong domain of each of the frameworks. Moreover, the solution to the shortcomings of the frameworks in addressing the complexities of modern healthcare data governance, particularly in decentralized systems, IoT-driven health data, and emerging AI applications, have been provided.  Furthermore, as advised, the integration of the frameworks as the potential solution is now discussed in the following words:  ***“Blockchain Integration as a Solution***  *The potential of blockchain in healthcare governance strongely focuses on findings and discussions around its application. Blockchain offers:*  ***Decentralization & Transparency****: It enables secure, immutable, and tamper-proof data records, reducing reliance on central authorities.*  ***Smart Contracts for Compliance****: Automates compliance by ensuring healthcare data is only accessed under predefined conditions.*  ***Improved Data Access & Security****: Provides controlled access through cryptographic mechanisms, aligning with GDPR and HIPAA requirements.*  ***Cross-border Data Sharing****: Facilitates regulatory-compliant and secure international data transfers, addressing the gaps in existing governance models”.*  ***(Literature Review)*** |
| Although the manuscript provides a good overview of using blockchain technology in healthcare, however, it is worth mentioning its integration into existing data governance frameworks. Perhaps deeper technical discussion with examples and case studies will be beneficial. | Thank you for mentioning this point. As advised, by integrating **technical examples, case studies, and regulatory analysis**, we have tried to present a **strong case for blockchain’s role in healthcare data governance** which advocates for **policy updates and future studies** to bridge the gap between **regulatory frameworks and emerging technologies** like blockchain.  We have tried to effectively achieve a technical discussion with examples and case studies through several pf the following key strategies:  **Use of Concrete Technical Examples**  We have presented real-world examples, such as MedRec (MIT’s blockchain-based EHR system) and Estonia’s eHealth Foundation, to illustrate blockchain’s role in secure data sharing, patient consent management, and data integrity. These cases demonstrate how blockchain aligns with HIPAA’s access control and GDPR’s principles of transparency and accountability while ensuring tamper-resistant health data management.  **Integration with Regulatory Frameworks**  We have tried to connect these case studies to HIPAA, GDPR, and ISO standards. It highlights blockchain’s encryption, decentralized storage, and audit trails, which enhance compliance with HIPAA’s privacy and security regulations. However, it also addresses challenges, such as blockchain’s immutability conflicting with GDPR’s right to erasure, suggesting off-chain storage and cryptographic techniques as potential solutions.  **Comparison with Existing Literature**  By referencing studies from Mettler (2016) and Winter & Davidson (2017), we emphasised that ISO and GDPR provide broad governance frameworks but lack healthcare-specific guidelines. In contrast, HIPAA’s patient data protection focus makes it more compatible with blockchain solutions. The study’s findings further support this, showing high HIPAA compliance benefits from blockchain adoption.  ***(Discussion)*** |
| Literature review is comprehensive, but it could be more beneficial if critical analysis of existing studies is done. Perhaps more focused discussion is required about how blockchain technology has been previously applied in healthcare data governance | As advised, a literature review has been revised by focusing more on the application of blockchain technology in healthcare data governance, previously. Moreover, blockchain is considered a potential solution for challenges in healthcare data governance. Key points related to blockchain in healthcare include:  **Decentralized Data-Sharing & Security**: The existing privacy frameworks (ISO, GDPR, HIPAA) struggle with modern decentralized data-sharing models. Blockchain, by design, offers a decentralized, tamper-resistant ledger, which can enhance security and privacy in healthcare data management.  **Integration with Emerging Technologies**: The discussion on IoT and big data governance suggests the need for robust frameworks to manage real-time data securely. Blockchain can play a role by ensuring secure data provenance and controlled access in IoT-driven healthcare environments.  **Data Access Control**: The literature stresses the importance of well-defined access control mechanisms. Blockchain-based smart contracts can enforce role-based access and authentication to ensure that only authorized personnel handle sensitive healthcare data.  ***(Literature Review)*** |
| Methodology is well explained, however, the rationale for selecting 250 participants and the specific cities could be better explained. It is good to include the detailed description of questions in the appendix | Thank you very much for bringing our attention to the shortcomings in methodology. We have now added more details in the methodology section regarding the rationale for selecting 250 participants and specific cities. The following text, which has now been added to the manuscript, will elaborate the rationale:-  *“The research focused on participants from Islamabad, Lahore, and Karachi, chosen for their diverse demographic profiles, advanced healthcare facilities, and varying levels of technology adoption. These cities represented both developed and developing health systems, allowing for a comprehensive assessment of data governance effectiveness in different contexts. A total of 250 participants were selected to ensure statistical validity, maintaining a 95% confidence level with a 5% margin of error while also considering practical limitations regarding participant availability.The participant pool included healthcare professionals, IT specialists, blockchain developers, administrators, and researchers, all of whom were directly involved in implementing and sustaining blockchain-based healthcare data platforms. Additionally, the 'other' category encompassed data analysts, compliance officers, and healthcare policy advisors, broadening the study’s perspective on governance structures. To ensure balanced representation across various organizational roles and levels within the healthcare industry, a stratified random sampling technique was employed. This approach strengthened the findings by incorporating diverse viewpoints from different professional backgrounds and organizational hierarchies.*  Moreover, as suggested, a detailed description of questions has now been added in the appendix**.**  ***(Methodology)*** |
| The results have been explained well, but it can be improved e.g. the findings of HiPAA is perceived as more effective than ISO and GDPR, delve deeper into why this might be the case. More statistical significance, particularly in relation to practical implications for healthcare data governance is required. | Thank you for mentioning this point. As advised, more statistical significance has been added by incorporating two new tables (Tables 7 & 8) to show the confidence Intervals & Mean Scores of Frameworks (ISO, GDPR, HIPAA) and ANOVA, results for Significance Testing respectively. The following text has been added:- *“Table 7 shows the mean scores and 95% confidence intervals for the efficiency of ISO, GDPR, and HIPAA frameworks on three key dimensions: Protection, Trust, and Security. The results show that HIPAA consistently records the highest mean values across all aspects, with a Protection mean 4.23, Trust mean 4.04, and Security mean 4.41, pointing to its better performance in maintaining data security and building trust over ISO and GDPR. The relatively small confidence intervals also imply the stability and reliability of the findings. Conversely, ISO demonstrates the lowest scores on all dimensions, indicating possible gaps in perceived effectiveness, especially when compared to GDPR and HIPAA.* TABLE 7 CONFIDENCE INTERVALS & MEAN SCORES OF FRAMEWORKS (ISO, GDPR, HIPAA) **Framework** \| **Protection Mean** \| **95% CI (Protection)** \| **Trust Mean** \| **95% CI (Trust)** \| **Security Mean** \| **95% CI (Security)**; **ISO** \| 3.45 \| [3.36, 3.54] \| 3.45 \| [3.37, 3.54] \| 3.61 \| [3.51, 3.72]; **GDPR** \| 3.81 \| [3.72, 3.90] \| 3.67 \| [3.57, 3.78] \| 3.90 \| [3.80, 3.99]; **HIPAA** \| 4.23 \| [4.13, 4.34] \| 4.04 \| [3.95, 4.13] \| 4.41 \| [4.32, 4.51] ANOVA Results for Significance Testing *“Table 8 shows the ANOVA results, which indicate statistically significant differences (p < 0.001) between the three frameworks for all variables. The large F-values for Protection (63.54), Trust (38.45), and Security (66.90) support that these differences are not a result of random variation. The largest variance is seen in the Security dimension, where HIPAA's performance is significantly different, affirming its excellent performance in protecting healthcare data. These results all reinforce HIPAA's strength as a framework for data governance, especially in situations that require high degrees of data security and trust.”* TABLE 8 ANOVA RESULTS FOR SIGNIFICANCE TESTING **Variable** \| **F-Value** \| **p-Value** \| **Interpretation**; **Protection** \| 63.54 \| p < 0.001 \| Significant differences between ISO, GDPR, and HIPAA.; **Trust** \| 38.45 \| p < 0.001 \| Significant differences in building trust among frameworks.; **Security** \| 66.90 \| p < 0.001 \| HIPAA shows the highest perceived security effectiveness. ***(Tests & Results)*** |
| The references are comprehensive and relevant, but there are few areas where more recent studies could be included, specifically the discussion around blockchain technology and its applications in healthcare. | As advised, several additional references have now been included in the manuscript in relation to the discussion on blockchain and its application in the healthcare sector. Some of them are as follows:-   - Danielyan, A. (2019). *Penetration Testing Essentials*. Wiley. - Easttom, C. (2018). *Computer Security Fundamentals* (3rd ed.). Pearson IT Certification. - Holt, T. J., Bossler, A. M., & Seigfried-Spellar, K. C. (2017). *Cybercrime and Digital Forensics: An Introduction*. Routledge. - Lee, A. J. (2020). *Offensive Security: Ethical Hacking Strategies and Techniques*. Packt Publishing. - Russo, J. (2018). *Cybersecurity and Cyberwar: What Everyone Needs to Know*. Oxford University Press. - Smith, R. E. (2020). *Elementary Information Security* (3rd ed.). Jones & Bartlett Learning.   ***(Throughout the manuscript and References)*** |
| Phrasing and grammatical errors that should be addressed. e.g. "The results revealed unsatisfaction for data governance frameworks..." should be rephrased to "The results revealed dissatisfaction with data governance frameworks..." The tables are well presented, but the bar chart on pg.19 could be improved for clarity. | Thank you for bringing our attention to the phrasing and grammatical errors. These have been removed throughout the manuscript for a better read. Also, we engaged a copyeditor who thoroughly reviewed the complete document and helped in improving the document, especially, in terms of phrasing and grammar.  ***(Throughout the Manuscript)***  Moreover, as advised, the bar chart has now been updated for more clarity. |
